# Influence of Heat Treatment of Electrospun Carbon Nanofibers on Biological Response

**DOI:** 10.3390/ijms23116278

**Published:** 2022-06-03

**Authors:** Jaroslaw Markowski, Marcel Zambrzycki, Wojciech Smolka, Agnieszka Panek, Maciej Gubernat, Paweł Czaja, Mateusz Marzec, Aneta Fraczek-Szczypta

**Affiliations:** 1 Laryngology Department, School of Medicine in Katowice, Medical University of Silesia in Katowice, Poniatowskiego 15, 40-055 Katowice, Poland; jmarkowski@sum.edu.pl (J.M.); wojciech.smolka@op.pl (W.S.); 2 Faculty of Materials Science and Ceramics, AGH University of Science and Technology, Mickiewicza 30 Av., 30-059 Krakow, Poland; zambrzycki@agh.edu.pl (M.Z.); maciej.gubernat@agh.edu.pl (M.G.); 3 Institute of Nuclear Physics, Polish Academy of Sciences, Radzikowskiego 152 St., 31-342 Krakow, Poland; agnieszka.panek@ifj.edu.pl; 4 Institute of Metallurgy and Materials Science, Polish Academy of Science Reymonta 25 St., 30-059 Krakow, Poland; p.czaja@imim.pl; 5 Academic Centre for Materials and Nanotechnology, AGH University of Science and Technology, Mickiewicza 30 Av., 30-059 Krakow, Poland; marzecm@agh.edu.pl

**Keywords:** electrospun carbon nanofibers (eCNF), carbonization, genotoxicity, crystallinity, chondrocytes, biomaterials, fluorescence microscopy, comet test, nitrile groups

## Abstract

The main aim of this study is to investigate the effect of fragmentation of electrospun carbon nanofibers (eCNFs) obtained at different temperatures, i.e., at 750 °C, 1000 °C, 1500 °C, 1750 °C and 2000 °C on the cellular response in vitro. In order to assess the influence of nanofibers on biological response, it was necessary to conduct physicochemical, microstructural and structural studies such as SEM, XPS, Raman spectroscopy, HRTEM and surface wettability of the obtained materials. During the in vitro study, all samples made contact with the human chondrocyte CHON-001 cell lines. The key study was to assess the genotoxicity of eCNFs using the comet test after 1 h or 24 h. Special attention was paid to the degree of crystallinity of the nanofibers, the dimensions of the degradation products and the presence of functional groups on their surface. A detailed analysis showed that the key determinant of the genotoxic effect is the surface chemistry. The presence of nitrogen-containing groups as a product of the decomposition of nitrile groups has an influence on the biological response, leading to mutations in the DNA. This effect was observed only for samples carbonized at lower temperatures, i.e., 750 °C and 1000 °C. These results are important with respect to selecting the temperature of thermal treatment of eCNFs dedicated for medical and environmental functions due to the minimization of the genotoxic effect of these materials.

## 1. Introduction

Due to their variety of forms, carbon materials have been studied in various fields of medicine for over 40 years. A particularly important form of synthetic carbon materials are carbon fibers obtained by pyrolysis in an inert environment of organic products such as rayon, polyacrylonitrile (PAN) and pitch [1]. For medical purposes, they are mainly obtained from polyacrylonitrile in a carbonization process at a temperature of about 1000 °C. However, these can also be treated at higher temperatures in order to obtain fibers with a high degree of crystallinity, namely graphite-like fibers. Optimizing the process of thermal treatment of carbon fibers causes these materials to change, not only in terms of structure but also in terms of the presence of functional groups and heteroatoms. These changes cause the fibers to differ in their mechanical, electrical and biological properties. Carbon fibers have been biologically tested many times and are used clinically in many areas of medicine [2]. Due to good electrical properties, carbon fibers have been used for neural recording as well as for stimulating cardiomyocytes. It has also been shown that carbon fibers influence the regeneration process in both soft and hard tissues [3]. Thanks to the modification of the fibers with bioactive particles such as hydroxyapatite or bioglass, the fibers improved their compatibility to bone tissue [4]. Carbon fibers were considered not only in the regeneration of bone tissue but also in the treatment of osteochondral defects formed on the articular surface of the patella [3,4,5,6,7]. In the 1980s, carbon fibers were used clinically as a scaffold to induce proliferation of tendon tissues or to repair ligaments [8]. However, the low shear strength of this material resulted in the formation of permanent degradation products of carbon fibers. For this reason, carbon fibers have not been approved by the Food and Drug Administration (FDA) to replace the anterior cruciate ligament. Carbon fibers are also the ideal reinforcement material in polymer composites, and this type of material has also been used to repair bone fractures in plate anastomoses and has been shown to heal wounds better than metal, allowing for stress to be distributed more evenly due to a lower Young’s modulus than metallic implants. The same property of carbon fiber composites was also used in research on the construction of hip joint stems. The controversy of the 1980s indicating the lack of biocompatibility of carbon fibers, thus showing their irritant effect and, consequently, the lack of FDA approval of the use of carbon fibers in the reconstruction of the anterior cruciate ligaments, led to a significantly reduced interest in this type of material used in direct contact with cells or tissues [9]. As shown by various scientists, the different opinions on the biocompatibility of carbon fibers can be explained by the use of different types of fibers that differ from each other in terms of structural, chemical and physical properties resulting from differences in their process parameters [2,3,10]. It has been shown that the cellular response to fiber carbon depends largely on the crystallinity of the material. Therefore, not all types of carbon are suitable for the treatment of tissues [3,11,12].

In recent years, carbon materials on the nanometric scale have attracted great interest in the fields of medicine and environmental protection. Here, we are talking mainly about carbon nanotubes, graphene and its derivatives, as well as carbon nanofibers obtained by electrospinning. Electrospun carbon nanotubes, on the one hand, may be an alternative to classic, micrometric carbon fibers and, on the other hand, are less controversial when used in biological applications than carbon nanotubes. The electrospinning method allows for the production of nanofibers of various diameters from several dozen to several hundred nanometers in the form of mats with random or directed/aligned fiber arrangements as well as with different densities and porosities [13]. On the downside, the appropriate selection of the parameters of the carbonization process leads to materials with a different degree of crystallinity and surface chemistry [14].

Electrospun carbon nanofibers (eCNFs) seem to be particularly interesting from the standpoint of creating scaffolds for the regeneration of hard tissues such as bone and/or cartilage [8,15]. In bone and cartilage engineering, scaffolds with appropriate mechanical properties, high biocompatibility, controlled biodegradation over time, as well as osteogenic and chondrogenic properties are desirable. A wide variety of materials such as ceramics, metals and polymers have been used to repair defects in bone and cartilage. However, none of these materials fully meet the requirements for substitutes for bone and cartilage. Metals and ceramics are not natural constituents of the human body, and polymers, in turn, have low mechanical strength and no osteogenic and chondrogenic induction properties. All this means that these materials have limited use in bone, cartilage or osteochondral tissue engineering [16]. Their unique structure and properties make synthetic carbon materials, in particular carbon nanofibers, promising candidates for tissue engineering applications [17].

Thus far, all research related to carbon nanomaterials for hard tissue regeneration has been carried out in vitro in cell cultures or in vivo in animals and has not yet been advanced into clinical trials. As with all materials, in particular nanomaterials, it is very important to recognize the influence of various factors such as concentration, dimensions, presence of impurities, functional groups, the degree of crystallinity (in the case of carbon nanofibers) and many others on avoiding cell cytotoxicity or genotoxicity. While many studies of carbon nanomaterials for cytotoxicity have already been carried out, many of the factors that regulate cell behavior are not well understood so far. It seems that one of the factors that may have a significant impact on the cytotoxicity of carbon nanofibers is the influence of the degradation products of these materials, which, as a result of mechanical action or degradation, e.g., enzymatic, can enter the body and irritate cells. Taking into account knowledge of the effects of degradation products of classic carbon fibers, it seems that research along this line is also worth pursuing in the case of nanofibers. When analyzing the products of degradation and fragmentation of carbon fibers, particular attention was paid to the size of these products and the degree of crystallinity. According to the current state of knowledge, the impact of carbon nanofibers degradation products, which may differ from each other in the degree of order and crystallinity, has not yet been described in the literature. Moreover, when examining the influence of degradation products on the cellular response, cytotoxic activity and, more rarely, genotoxic activity, are taken into account. 

The structure of the cell as well as its functions are encoded in genes, i.e., in chromosomes consisting of helixes of double DNA strands. Any change in an established DNA structure is called a mutation, and the factor causing it is called a genotoxin or genotoxic agent. Genotoxicity assessment seems to be particularly important in the case of materials on a nanometric scale. Intensive genotoxicity research indicates that two types of genotoxicity can be considered for nanomaterials, namely primary (direct or indirect) or secondary genotoxicity. Direct genotoxicity results from the physical contact of a nanoparticle with DNA in the cell nucleus. This interaction can lead to DNA damage such as ruptures, distortions, and damage to chromosomes. The primary indirect mechanism is due to nanomaterial-induced reactive oxygen species (ROS) that lead to oxidative stress or nanomaterial-induced inactivation of enzymes associated with DNA repair [18,19,20]. Secondary genotoxicity is a result of the action of the phagocytic cells present during inflammation, which, by releasing pro-inflammatory cytokines and other substances, indirectly influence the oxidative stress. Numerous studies have shown that the genotoxicity of nanoparticles depends not only on the exposure time and concentration of nanoparticles but also on their size, shape, surface properties and chemical composition [21,22,23,24]. 

The aim of this study was to determine the effect of degradation products and fragmentation of eCNFs obtained at different temperatures, i.e., at 750 °C, 1000 °C, 1500 °C, 1750 °C and 2000 °C on the cellular response of chondrocytes in vitro. The assessment used the comet test to examine the viability of cells and, above all, the impact of fragmented nanofibers on genotoxicity. Special attention was paid to the degree of crystallinity of the nanofibers, the dimensions of the degradation products and the presence of functional groups on their surface. In order to assess the influence of these factors on the potential genotoxicity and behavior of the chondrocyte cell line CHON-001 in contact with these materials, all samples were subjected to detailed structural, microstructural and physicochemical analysis.

## 2. Results and Discussion

### 2.1. Physicochemical, Microstructural and Structural Characterization of eCNFs 

**Figure 1:**
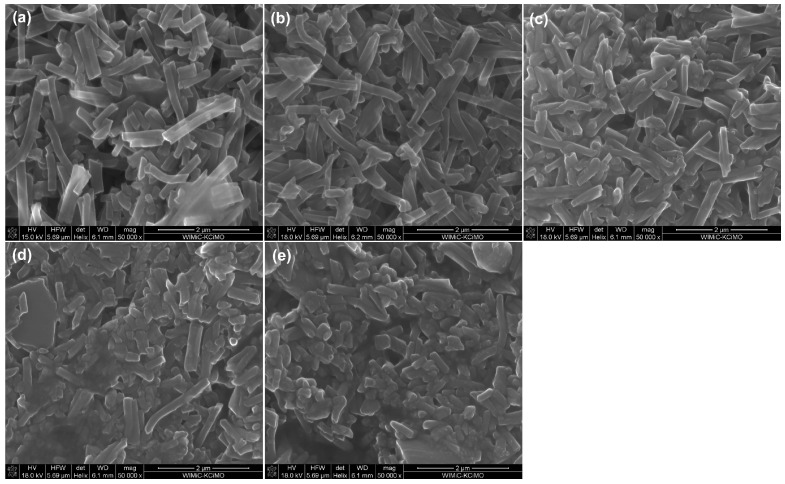
SEM microphotograph of fragmented eCNFs (**a**) eCNF750, (**b**) eCNF1000, (**c**) eCNF1500, (**d**) eCNF1750 and (**e**) eCNF2000.

Based on the scanning electron microscopy (SEM) results of carbon nanofibers, it can be observed that each type of material is degraded, as evidenced by a change in average fiber length. In terms of microstructure these fibers do not significantly differ from each other (Figure 1). 

**Figure 2:**
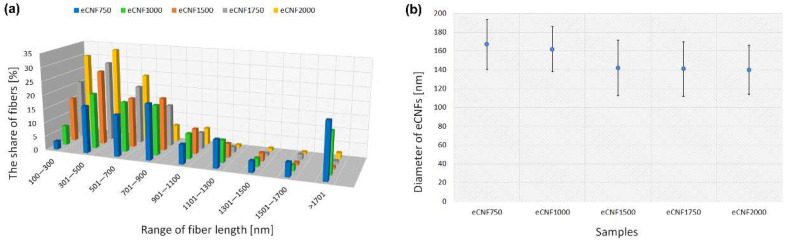
Length distribution of eCNFs (**a**) and average values of nanofiber diameters (**b**).

However, some differences can be observed in the length of the eCNF fragments. Using the ImageJ program, 100 nanofiber length measurements were made for each of the samples. The results obtained are presented in the graph below (Figure 2A) in the form of histograms showing the fiber length distribution. When analyzing the length distribution of nanofiber fragments, some differences between the samples can be observed. In the case of samples carbonized at low temperatures, i.e., 750 °C and 1000 °C (eCNF750 and eCNF1000), the share of nanofibers of shorter lengths, i.e., in the ranges of 100–500 nm as well as 500–900 nm, is relatively low compared with the share obtained at the temperatures of 1750 °C and 2000 °C. For these low-temperature samples, a greater share of nanofibers with lengths of over 1000 nm or even 1700 nm was observed compared with eCNFs subjected to thermal treatment at higher temperatures (eCNF1750 and eCNF2000). Lengths in the 100–700 nm range dominate in the higher temperature samples. The differences, especially between the eCNF750 and eCNF2000 samples, are most likely due to the degree of crystallinity of the samples. The higher the degree of crystallinity, the more brittle the samples, which may be manifested by the presence of a dominant number of fiber fragments of shorter lengths (eCNF2000). The more amorphous the nanofibers are, the less brittle they are, and therefore, longer length fiber fragments (eCNF1000) can be expected, as was observed in this study.

Additionally, using the ImageJ program, 100 measurements of nanofiber diameters were made for each of the samples. The results obtained are presented in a diagram (Figure 2B). The analysis of the change in the diameters of eCNF indicates a noticeable tendency towards a decrease in diameter when the thermal treatment of nanofibers is increased. The greatest decrease is observed up to a temperature of 1500 °C. Compared with the samples obtained at a temperature of 750 °C, it amounts to approx. 15%. Further thermal treatment does not actually affect the decrease in the diameter of nanofibers, which is related to the fact that the greatest changes in both structural arrangement and removal of non-carbon elements, affecting a noticeable decrease in weight and dimensions, occur at a temperature of between 1500 °C and 1700 °C. It should also be remembered that polyacrylonitrile is a precursor of carbon materials that does not undergo the graphitization process, so further thermal treatment process will have a limited impact on the ordering of the structure of this material but will cause a noticeable change in the geometric dimensions of the material.

In order to assess the structural parameters of the tested carbon nanofiber samples, Raman spectroscopy was performed. There are two main characteristic bands in carbon materials, namely the G and D bands [25,26,27]. The G band corresponds to the C–C radial stretching of sp^2^ carbon and is characteristic of well-ordered carbon structures. The D band corresponds to disordered carbon, and it is related to the presence of structural defects in the carbon structure and to the presence of heteroatoms and dangling bonds. The relative intensities from the D to G bands (I_D_/I_G_) are used as a parameter to monitor the structural ordering, the presence of defects in the carbon structure, and the purity and degree of functionalization of carbon materials including eCNF. A high I_D_/I_G_ ratio indicates the presence of defects inside the carbon layers, while a low ratio indicates high structural ordering and a small number of structural defects in carbon materials. Based on this parameter, using Formula (1) (Cançado equation), the lateral sizes of graphitic domains La were estimated. 

**Figure 3:**
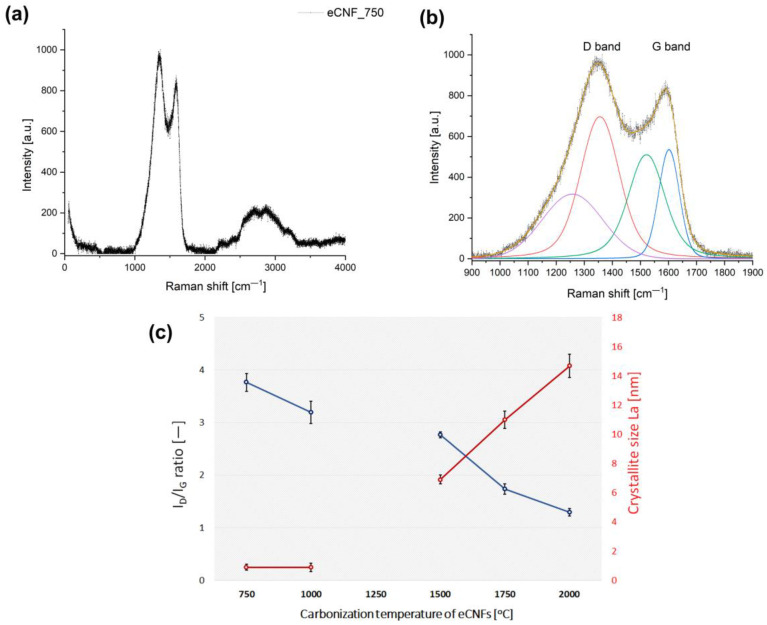
Raman spectrum for the eCNF_750 sample in full range (**a**) and the Raman spectrum of the same sample for the range of primary D and G bands subjected to deconvolution (**b**). Two parameters I_D_/I_G_ and La extracted from the Raman spectra and estimated from Cançado equation for CNFs obtained at different temperatures (**c**).

The results of the research carried out with the use of Raman spectroscopy clearly show an increase in the ordering of the structure of carbon nanofibers with an increase in the temperature of the thermal treatment, which is manifested by a decrease in the I_D_/I_G_ parameter (Figure 3). Thus, the results obtained are consistent with the assumptions and the theory of the influence of temperature on the re-arrangement of the carbon structure towards a graphite-like material. At the same time, an increase in the fiber carbonization temperature influenced an increase in the size of crystallites (La), which is also consistent with the theory. 

The vast majority of volatile products are released in a temperature range between 200 and 1000 °C. The evolved gases include HCN, H_2_O, O_2_, H_2_, CO, NH_3_, CH_4_, high molecular weight compounds and miscellaneous tars [28]. As the temperature of the PAN nanofibers increases, the turbostratic carbon phase is formed [14,29]. This phase appears at around 1500 °C and is well oriented in the fiber direction but still has many tetrahedral carbon-type crosslinks between the graphite-type carbon layers. The final step of thermal treatment of nanofibers based on PAN is at temperatures in the range of 2000–3000 °C, known as the graphitization process. At these temperatures the order and orientation of the small turbostratic crystallites occurs in the direction of the fiber axis [29]. 

**Figure 4:**
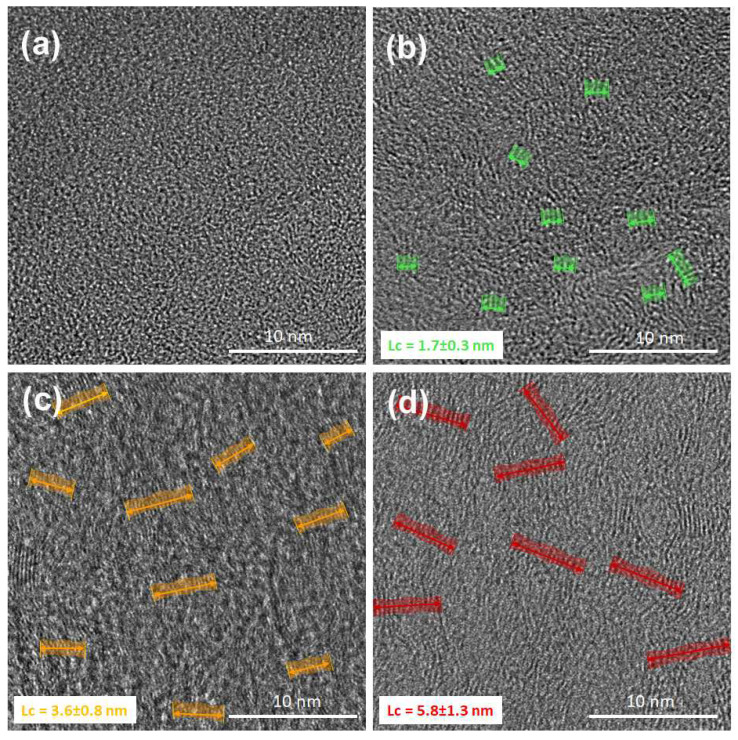
HRTEM micrographs of the transverse sections: (**a**) eCNF750, (**b**) eCNF1000, (**c**) eCNF1750 and (**d**) eCNF2000.

In the literature, on the basis of various methods such as X-ray diffraction, SEM, Raman scattering and transmission electron microscopy (TEM), various models of microstructure of carbon fibers based on PAN are presented [30,31,32,33,34]. The models are not consistent with each other, which makes the situation unclear [35]. However, the increase in the temperature of thermal treatment of PAN nanofibers causes the structure to be organized (Figure 4). Carbon nanofibers obtained at the lower temperatures, especially at 750 °C but also at 1000 °C have a strongly disordered, amorphous structure that was observed both using Raman spectroscopy (Figure 3) and high-resolution transmission electron microscopy (HRTEM) (Figure 4a,b). The increase in temperature of the thermal processing of carbon nanofibers indicates that a turbostratic structure is formed by the wrinkled and entangled crystallites composed of small graphitic basal planes. The formation of these areas of order is observed closer to the surface of the nanofiber, i.e., in the skin region of the nanofiber. This is particularly evident in the eCNF2000 nanofibers (Figure 4d). Based on the HRTM lattice image of nanofibers thermally treated at 1750 °C and 2000 °C, it is difficult to clearly show the crystallite dimension, especially the width perpendicular to the nanofiber axis (La) in the transverse sections of carbon nanofibers because it is difficult to measure La as the graphitic basal planes are often twisted or folded and crystallites in the nanofiber do not have a regular shape. However, one can attempt to approximate the thickness of the crystallites (Lc parameter). It was impossible to define this parameter for nanofibers carbonized at 750 °C, and the structure of this material is clearly amorphous. Some ordering can already be observed for the eCNF1000 sample. The value of the Lc parameter determined for this sample is 1.7 ± 0.3 nm (Figure 4b). The increase in the size of crystallites (parameter Lc) for the eCNF1750 and eCNF2000 samples confirms the ordering of the nanofiber structure with the increase in the temperature of the thermal treatment. The mean values of the Lc parameter for the eCNF1750 and eCNF2000 samples are 3.6 ± 0.8 nm and 5.8 ± 1.3 nm, respectively (Figure 4c,d).

The assessment of the chemical composition of the surface of the fragmented eCNFs was carried out using the X-ray photoelectron spectroscopy (XPS) method. This analysis showed the presence of three elements in the structure of nanofibers, namely carbon, oxygen and nitrogen. Table 1 shows the chemical composition of the eCNFs after various thermal treatments. 

The dominant element in each type of nanofiber is, of course, carbon (C1s), the atomic content of which changes with increasing temperature of thermal treatment. In addition to carbon the eCNFs also contain oxygen (O1s) of which the concentration decreases with increasing carbonization temperature (from 3.2% for eCNF750 to 0.4% for eCNF2000). The situation is similar with nitrogen (N1s), which is only observed in the eCNF750 and eCNF1000 samples, i.e., carbonized at lower temperatures. On the other hand, no nitrogen was observed in the carbonized samples at the temperatures of 1500 °C, 1750 °C and 2000 °C. The increase in carbon concentration in the eCNF samples with the increase in the carbonization temperature is a natural phenomenon because the main goal of thermal treatment is to increase the share of carbon in carbon-bearing materials while removing all non-carbon components [36,37]. 

**Figure 5:**
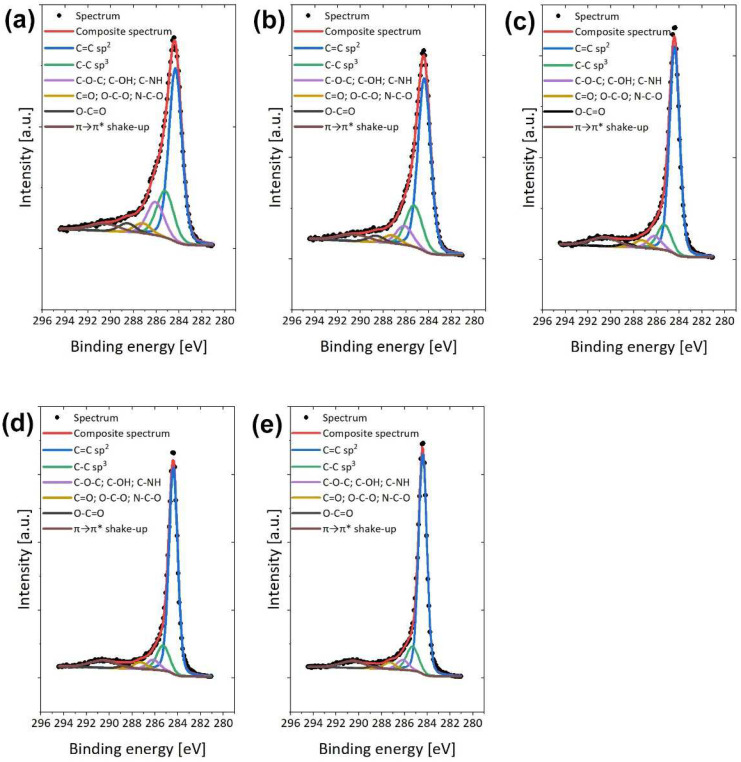
XPS spectra of carbon nanofibers carbonized at different temperatures. Deconvoluted C1s peaks at (**a**) eCNF750, (**b**) eCNF1000, (**c**) eCNF1500, (**d**) eCNF1750 and (**e**) eCNF2000.

The XPS method allowed not only for the determination of the elemental composition of the sample but also for measurement of the concentration of the chemical bonds on the eCNFs’ surfaces after different heat treatment temperatures. The C1s spectra for all samples were fitted with five components, of which the first lies at a binding energy of 284.4 eV, which indicates C=C (sp^2^) type bonds; the second lies at 284.9 eV, which indicates C-C (sp^3^) bonds; peak 3 lies at 286.2 eV, pointing out the existence of C-O-C, C-OH, and/or C-NH bonds; peak 4 lies at 287.5 eV, indicating the presence of C=O, O-C-O, and/or N-C-O bonds; peak 5 lies at 288.6 eV, showing O-C=O type bonds; and the component from the π→π* satellite lies at 290.1 eV [38,39,40]. The share of the π→π* shake-up was in direct correlation with the graphitic character of the samples. Deconvolution of the C1s band for eCNF750, eCNF1000, eCNF1500, eCNF1750 and eCNF2000 is presented in Figure 5.

**Figure 6:**
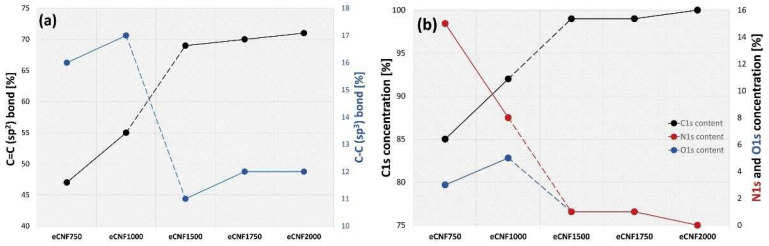
Comparison of the percentage of C=C (sp^2^) and C–C (sp^3^) bonds (**a**) and concentration of C1s, N1s and O1s (**b**) for eCNFs carbonized at different temperatures.

The sp^2^ content calculated from the C1s spectra indicates a significant increase in the sp^2^ hybridized carbon with the increase in thermal treatment of nanofibers. At the same time, as the carbonization temperature increases, the percentage of sp^3^ (C–C) bonds related to the presence of defects in the carbon structure decreases. A summary of the sp^2^ and sp^3^ carbon atoms occurring in the form of C=C and C–C bonds, respectively, is shown in Figure 6a.

The increase in the percentage of carbon in sp^2^ hybridization shows a clear tendency towards the gradual development of the planar structure of π-conjugated carbon and the reduction in the defect density with the increase in the temperature of eCNFs carbonization. The increase in sp^2^ percentage clearly indicates the ordering of the nanofiber structure with the increase in temperature of thermal treatment, which is also confirmed by the results of Raman spectroscopy and HRTEM (Figure 3 and Figure 4).

In the eCNFs samples, two oxygen (O1s) bonds were distinguished: at 530.7 eV, corresponding to group C=O, and at 532.8 eV, corresponding to functionalized oxygen-containing groups, such as C–OH or C–O–C [41]. The concentration of oxygen groups in eCNFs is small, and those that are there are associated particularly with C–OH or C–O–C bonds. Moreover, their content decreases with the ordering of the carbon structure in nanofibers, i.e., 2.4% for eCNF750 and 0.4% for eCNF2000 (Figure 6b, blue line). 

The presence of nitrogen in eCNF samples carbonized at lower temperatures (eCNF750 and eCNF1000) is a result of residues of nitrile groups (C≡N) present in the polymer precursor of nanofibers, namely PAN. During the stabilization process of polyacrylonitrile nanofibers, nitrile groups are subject to systematic disappearance accompanied by the formation of either imide and amino groups, or it may indicate the formation of volatile compounds resulting from polymer decomposition, containing nitrogen atoms, e.g., NH_3_ (ammonia) and HCN (hydrogen cyanide). To confirm the presence of nitrogen groups, the deconvolution of the N1s band was performed, although the XPS spectra were not included in the article. This analysis confirms that the presence of nitrogen in carbonized samples at lower temperatures is related to the presence of C-N bonds and/or –NH- in aromatic ring and form –NH3+ compounds or –N(CH3)3+ groups. These groups, however, disappear completely with increasing carbonization temperatures and, in samples eCNF1500, eCNF1750 and eCNF2000, are no longer measurable (Figure 6b, red line). 

**Figure 7:**
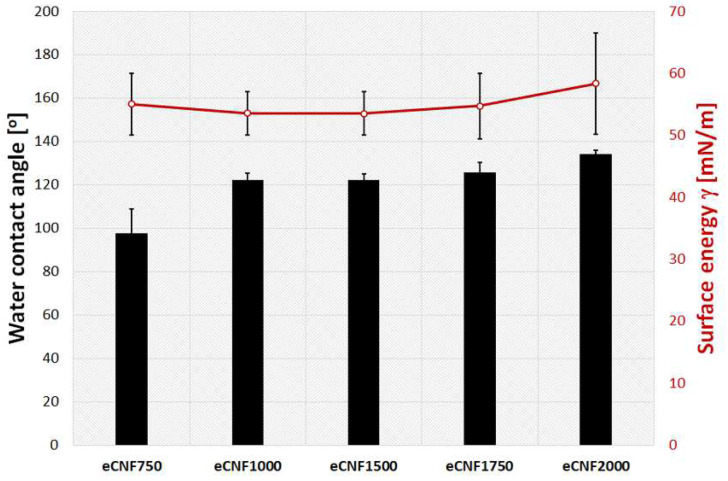
Water contact angle values and surface energy for eCNFs after carbonization at different temperatures.

Wettability is one of the fundamental properties of solid-state surfaces that characterizes their degree of wetting by a liquid droplet. Wettability can be quantified through the contact angle (𝜃), which is the angle between the solid–liquid and liquid–gas interfaces. Surface wettability is one of the most frequently defined parameters influencing the biological response of cells. The presence of functional groups on the material’s surface, in this case, eCNF, has a decisive influence on the degree of surface wettability. The contact angle test results and their images are shown in the diagram below (Figure 7) and in the Supplementary Materials (Figure S1), respectively. 

In general, all carbon materials, including eCNF, have a hydrophobic surface due to their structure, where carbon exists almost exclusively in the form of aromatic, non-polar sheets, so their interaction with polar molecules such as water is very low [42]. Graphite, as a model material, is assumed to have contact angle values in the range from 75° to 95° [43,44,45]. However, depending on the form, dimensions, structure or the degree of functionalization, carbon materials can have different values, both more hydrophilic and superhydrophobic [46,47]. The contact angle of the samples increases with the temperature of the thermal treatment of eCNFs. The eCNF750 nanofibers have the lowest value of contact angle 𝜃 = 97.8 ± 10.9°, whereas the eCNF2000 nanofibers have the highest contact angle 𝜃 = 134.3 ± 1.7°. The values of the contact angles for the remaining samples are within the range of approximately 𝜃 = 120–125° (Figure 7). The increase in hydrophobicity of carbon nanofibers with increasing temperature results from the removal of functional groups, mainly oxygen groups, from the surface of the nanofibers as well as the removal of heteroatoms from the carbon structure. The decrease in oxygen-related functional groups for eCNFs carbonized at higher temperatures (eCNF1500, eCNF1750 and eCNF2000) is confirmed by the results of the XPS tests (Table 1 and Figure 6b). The contact angle values for all tested eCNFs are high, especially for those carbonized at higher temperatures. Such high contact angles (above 120°) may also result from the porosity and roughness of carbon nanofibers and be consistent with the model proposed by Cassie and Baxter (no droplet penetration) assuming that the bottom of the droplet partially wets the rough substrate due to the existence of air pockets in between the microstructures [48,49]. While some differences in the surface wettability of different eCNFs was observed, the value of the total surface energy for all analyzed samples is at a similar level, in the range from 53 mN/m to 58 mN/m. 

The zeta potential at the boundary of carbon nanofibers and phosphate-buffered saline (PBS) was assessed by measuring the electrophoretic mobility of the particles in solution based on a light scattering analysis. According to standard [50], if the absolute value of ξ is less than ± 25 mV, the particles start to agglomerate because the forces related to repulsion of the particles are not strong enough to resist the van der Waals attraction forces between the particles [51,52]. The presented results indicate that, for all eCNF samples, the ξ potential values are below the value of ± 25 mV, which suggests a rather low stability of the eCNF degradation products in the PBS solution (Table 2). Minimal differences in zeta potential were observed between these materials.

### 2.2. Biological Evaluation of eCNFs 

**Figure 8:**
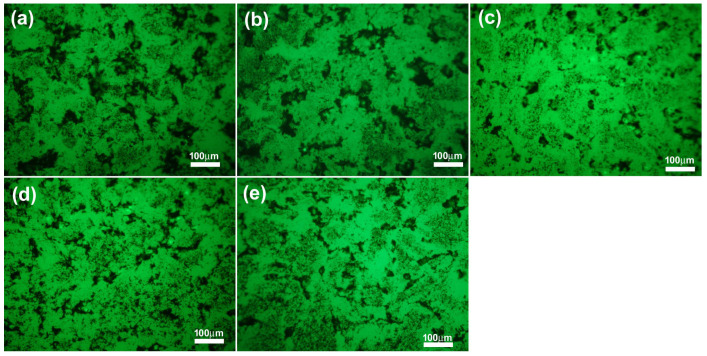
Fluorescence micrographs for (**a**) eCNF750, (**b**) eCNF100, (**c**) eCNF1500, (**d**) eCNF1750 and (**e**) eCNF2000 showing the behavior of fragmented eCNFs in the culture medium forming agglomerates of various shapes and sizes.

In vitro studies were carried out on one type of cell, namely the chondrocyte cell lines CHON-001. The tests of the morphology of cells in contact with eCNFs were investigated using staining with calcein and propidium iodide. This method also allowed us to determine the presence of the potential dead cells, which were stained with propidium iodide in the tested culture. These studies were performed using a fluorescence microscope. Fluorescence microscope observations were also made to assess the behavior of the fragmented eCNFs in contact with cells in the culture medium (Figure 8).

As can be seen, the largest agglomerates are formed in the case of carbonized carbon nanofibers at lower temperatures, i.e., 750 °C and 1000 °C. This may be related to the dimensions of these eCNFs; for these materials, the longest lengths of the fragmented eCNFs were observed, which was confirmed both in the SEM microphotographs (Figure 1a,b) and by the analysis of the CNF length distribution (Figure 2a). As mentioned earlier, the smaller size of the degradation products of the fibers thermally treated at higher temperatures results from their higher crystallinity and thus a greater tendency to crumble, which is observed in the case of graphitized fibers. For both the eCNF750 and eCNF1000 samples, much greater fraction fibers with lengths of over 1000 nm or even 1700 nm were observed compared with other nanofibers (Figure 2a). The results of the research on the zeta potential of nanofibers in PBS border on the standard values, indicating good stability of these matrices in solution (Table 2). Thus, eCNF fragments in PBS solution should tend to agglomerate, as shown in the photos obtained from fluorescence microscopy (Figure 8). An additional factor that may imply the formation of agglomerates in cell culture is the presence of various components of the culture medium, such as hormones, proteins, vitamins or growth factors.

**Figure 9:**
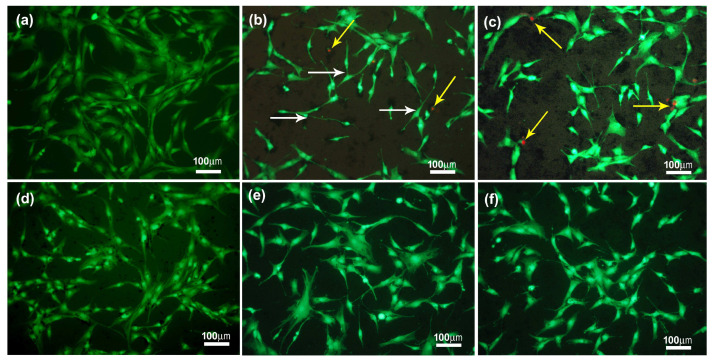
Merged microphotographs of human chondrocyte cell line (CHON-001) stained with calcein AM/propidium iodide grown on control (PS) (**a**) and eCNF750 (**b**), eCNF1000 (**c**), eCNF1500 (**d**), eCNF 1750 (**e**) and eCNF2000 (**f**).

The next step was to evaluate the behavior of chondrocytes in contact with the tested material. The chondrocyte morphology was assessed after 72 h of culture in contact with eCNF (Figure 9). The bottom of the polystyrene culture plate was used as a control. In order to assess the morphology of chondrocytes in vitro as well as to assess the viability of cells in contact with eCNFs, observations were made using fluorescence microscopy with the use of two dyes, i.e., calcein and propidium iodide. The tests were performed for one concentration of nanofibers, i.e., 42 µg/mL after 72 h of culture. The results for the control sample are shown in Figure 9a. It shows that the cells are in good condition, well flattened and spreading and have the shape typical of the CHON-001 cell line in vitro culture. No dead cells were observed in the images below. For all tested materials, no significant changes in the morphology of the tested cells were observed regardless of the conditions of the thermal processing of the nanofibers (Figure 9b–f). In general, the cells have a similar morphology to that of the control sample, are well spread, connect to each other and generally show no unexpected features. Some subtle changes in the morphology of chondrocytes can be seen only in contact with eCNF750 nanofibers (Figure 9b white arrows), where a certain fusiform and elongated shape of the cells can be observed, suggesting that, in contact with these materials, the cells flatten weaken or slow down. In general, only single dead cells could be seen in the samples carbonized at lower temperatures, i.e., eCNF750 and eCNF1000 (Figure 9b,c, yellow arrows).

**Figure 10:**
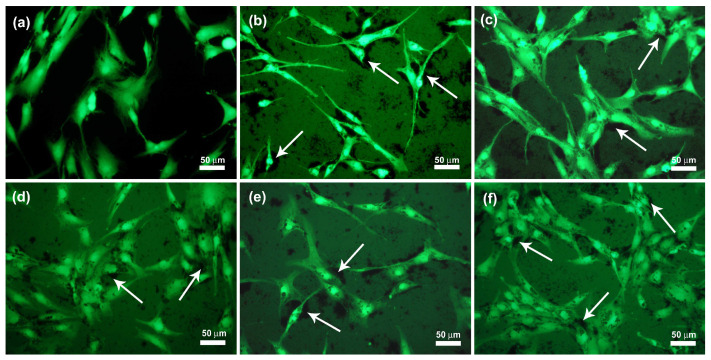
Merged microphotographs of human chondrocyte cell line (CHON-001) stained with calcein AM/propidium iodide grown on control (PS) (**a**) and agglomerates of eCNF750 (**b**), eCNF1000 (**c**), eCNF1500 (**d**), eCNF 1750 (**e**) and eCNF2000 (**f**).

In this study, it was also shown that chondrocyte cells do not avoid nanofiber agglomerates; on the contrary, they seem, in many cases, to prefer adhesion at the sites of eCNF agglomerates (Figure 10, arrows). Additionally, the morphology of these cells in contact with the nanofibers appears to be correct. One of the possible factors affecting the adhesion of cells to the surface of nanofiber agglomerates may be surface area (determined by the BET method), which for eCNF, is on average 20 m^2^/g (the results presented in our previous article [53]), as well as the dimensions of the nanofibers themselves, which are similar in size to some cell structures present in the cell membrane, such as membrane receptors. The large surface area is related to the presence of a greater number of active sites on the surface of the material (in this case nanofibers), which contributes to easier and faster adsorption of proteins and thus cell adhesion. In addition, the biomimetic nature of the eCNFs related to the similar dimensions to membrane receptors may have a positive influence on the adhesion of cells to their surface [54,55,56].

The shape of the cell is comparable with the shape of chondrocytes on the surface of the control sample (Figure 10a). Only in the case of the eCNF750 sample do the cells that adhere to the surface of the agglomerates have a spindle-like shape; on the remaining samples the cells are well spread, connect with each other and do not differ morphologically from the control sample. Thus, cell viability, determined on the basis of calcein and propidium iodide staining tests, indicates the absence of disturbing signals from the morphology, degree of crystallinity, the presence of agglomerates or the surface chemistry of carbon nanofibers after 72 h in culture. Only some differences from the other samples can be observed for the eCNF750 sample, where the shape of the cells in contact with this material is more elongated than for the others (Figure 10b).

**Figure 11:**
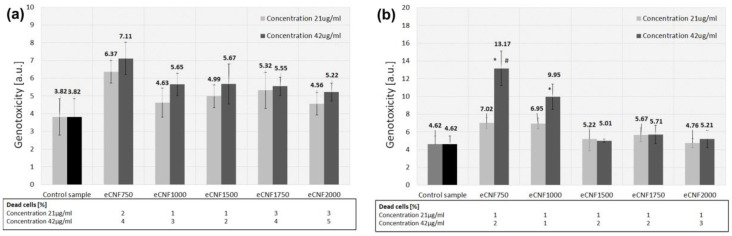
Results of genotoxicity of eCNFs in contact with the CHON-001 cell line using a comet assay after 1h (**a**) and 24 h (**b**) of culture. Two-way analysis of variance (ANOVA; control, concentration of eCNFs) with Student’s t-test post hoc test was used for statistical analysis. * *p* < 0.05 vs. PS for each time point; # *p* < 0.05 vs. eCNF concentration for each time point.

In addition to testing cell viability or the toxic effect of a potential biomaterial on the cellular response, an important factor that should also be taken into account is the assessment of the impact of the tested material on cellular genotoxicity. The structure of the cell as well as its functions are encoded in genes, i.e., in chromosomes consisting of helixes of double DNA strands. Any change in an established DNA structure is called a mutation, and the factor causing it is called a genotoxin or genotoxic agent. Genotoxicity assessment seems to be particularly necessary to perform in the case of materials on a nanometric scale. The genotoxicity assessment was performed using the comet test. It is one of the index tests that allow for the detection of DNA damage (single- and double-strand breaks, and specific DNA damage). The comet test is the most frequently used method of testing the genotoxicity of nanomaterials [21,57,58]. Genotoxicity testing was performed for two eCNF concentrations (21 µg/mL and 42 µg/mL) at two time intervals, i.e., after 1 h and 24 h in contact with the chondrocyte cell line. The chart (Figure 11a) shows the level of DNA damage for all tested eCNFs after 1 h of incubation. None of the samples showed a statistically significant increase in DNA damage compared with the control samples (well of the polystyrene (PS) culture plate). These results suggest that carbonized and graphitized carbon nanofibers at temperatures from 750 °C to 2000 °C do not damage DNA after 1 h of incubation. There are also no statistically significant differences between the applied concentrations of the tested compounds. Figure 11b shows the level of DNA damage for test compounds after 24 h of incubation. The eCNF750 (threefold) and eCNF1000 (twofold) samples showed statistically significant higher DNA damage level from the control sample. However, there was no statistically significant increase in cell death compared with the controls. It remained at a similar level to the samples after 1 h of incubation—maximum 5% of the dead cells. For the eCNF750 sample, a significant increase in genotoxicity was also observed with increasing concentration. Namely, the higher concentration (42 µg/mL) resulted in an almost twice as high level of genotoxicity compared with the lower concentration (21 µg/mL). These results indicate that the samples subjected to heat treatment at higher temperatures—eCNF1500, eCNF1750 and eCNF2000—showed no genotoxicity compared with samples carbonized at lower temperatures. Since DNA fragmentation is also associated with the process of cell death (apoptosis), false-positive results and the classification of cytotoxic compounds as genotoxic may occur if cytotoxicity is not an integral part of the genotoxicity test. Therefore, cells with more than 90% of DNA migration out of the nucleus are classified as dead. An analysis of the number of dead cells based on the comet test was performed (Figure 11a,b). After both 1 h and 24 h of cell culture, no significant increase in cell death was observed for any of the tested samples of carbon nanofibers.

When examining the influence of nanomaterials on genotoxicity, several factors are mentioned that may influence the level of genotoxicity. Apart from the dose of nanomaterials and the contact time of cells with the tested material, other factors determining genotoxicity include size, surface properties, chemical composition and shape [21,22,24,59]. In the case of CNF, previous scientific studies have reported an increase in the amount of DNA damage assessed with the γ-H2AX test [60] or disturbances in the mitotic spindle observed with the micronucleus test [61]. Electrospun carbon nanofibers are materials that do not dissolve in a biological environment. It is recognized that two processes are involved in the induction of genotoxic effects by low solubility nanoparticles: primary genotoxicity, which depends on the intrinsic activity of the particles (i.e., particle size, shape, particle uptake and the presence of mutagens carried with the particles), while secondary genotoxicity is related to particle-induced inflammation events. It is known that the ability of particles to produce ROS plays a major role in primary genotoxicity. These reactive species can arise on the surface of the particles or be associated with the presence of chemicals released by the particles or catalysts such as iron [61,62,63]. In the case of the tested nanomaterials, there is no problem with contamination with metallic particles, which are the residue of catalysts, as is the case in carbon nanotubes (CNT) or nanofibers obtained in the gas phase (VGCNF—vapor grown carbon nanofiber), so one of the main factors inducing genotoxicity in the tested samples does not occur. The genotoxic effect was observed in the case of the eCNF750 and eCNF1000 samples, specifically in the case of low-carbonized samples in contact with the cells for a longer period of time (24 h) and with a higher concentration of nanofibers (42 µg/mL). For the samples thermally processed at higher temperatures, no effect of these nanofibers on the fragmentation of the DNA chain of cells was observed, neither in the case of a higher concentration nor in the case of a longer culture time. The main factors differentiating the eCNF 750 and eCNF1000 samples from the eCNF1500, eCNF1750 and eCNF2000 samples are the length of the fragmented nanofibers; the presence of functional groups, especially oxygen and nitrogen groups; and the degree of crystallinity. Therefore, these three factors should be considered as potential influences on genotoxicity in contact with cells. 

During studies conducted on classic carbon fibers (microfibers), a significant influence on cytotoxicity, including carcinogenicity (secondary genotoxic effect), was seen in the degree of crystallinity. The more crystalline the samples of carbon fibers and the more ordered their structure, the worse the biological response [6]. The authors of these papers indicated that carbon fibers with higher crystallinity and a better-organized graphite structure were assimilated by the body with more difficulty, and small particles coming from these materials were found in the regional lymph nodes [6]. Furthermore, in the case of carbon nanofibers obtained by electrospinning, an increase in genotoxicity was observed in the case of nanofibers with lower crystallinity and a high degree of structural disorder. In the case of the tested samples, the increase in crystallinity did not affect genotoxicity or cell viability. Our previous studies also indicated that carbon nanofibers obtained at 1000 °C cause DNA damage (studies performed on normal human skin fibroblasts from the cell line CCL 110) compared with the controls [64]. The main reason was the lack of chemical functionalization of the surface and the high hydrophobicity of this type of material. The results published in the same article show a positive effect of surface functionalization through the increase in oxygen functional groups on both genotoxicity and cytotoxicity. The latest research results presented in this paper expand on the knowledge about the influence of carbon nanofibers on both cell viability and, most importantly, on genotoxicity and prove that not only surface functionalization but also carbonization of samples at higher temperatures, i.e., above 1000 °C (we observed it at 1500 °C) has positive effects on the cellular response. Therefore, if, on the one hand, the presence of oxygen functional groups (as confirmed in our earlier publication [64]) and, on the other hand, the increase in the degree of crystallinity of the samples do not induce genotoxic activity, there must be another factor related to carbon nanofibers carbonized at 750 °C and 1000 °C that produces this effect. Another factor that distinguishes the eCNF750 and eCNF1000 samples from the eCNF1500, eCNF1750 and eCNF2000 samples is the absence of nitrogen as a residue of nitrile groups present in the PAN precursor in the samples carbonized at the temperatures of 1500 °C, 1750 °C and 2000 °C. As the results of the XPS tests show, the greatest amount of nitrogen is present in the eCNF750 sample and slightly less in eCNF1000 (Table 1), which is related to the presence of C-N bonds and/or -NH- in aromatic rings and form -NH^3+^ compound groups. According to the data in the literature, the amino and nitrile groups may adversely affect the cellular response, including genotoxic effects, causing chromosomal aberrations and the exchange of sister chromatids [65,66,67]. Thus, it seems that the presence of functional groups as a residue after the removal of nitrile groups from the polyacrylonitrile structure or the presence of other nitrogen-containing groups as a product of the decomposition of nitrile groups may influence the biological response, causing genotoxicity. It appears that a potential genotoxicity mechanism may be based on covalent adducts with DNA guanines [68]. The second route is through reactions with P450 enzymes and peroxidases causing the formation of ROS and induced lipid peroxidation, which may also lead to mutations in DNA [69,70]. The research on the influence of the temperature of the thermal treatment of electrospun carbon nanofibers and the observed structural and physicochemical changes in them in relation to the cellular response, in particular the genotoxicity carried out by means of the comet test, are innovative. The results obtained may also be an indication of how, i.e., at what temperatures, the thermal treatment of PAN nanofibers should be carried out in order to obtain eCNFs with good biocompatibility, lack of genotoxicity, both in terms of future medical applications and environmental protection.

## 3. Materials and Methods

Carbon nanofibers (CNF) were obtained by the thermal treatment of polyacrylonitrile (PAN) nanofibers produced by electrospinning. A custom-made set-up with rotary collector was used for the electrospinning process. Nanofibers were spun using the following conditions: U = 11 kV; nozzle diameter dn = 1.1 mm; collector-nozzle distance s = 40 mm; t = 30 min; and gravitational outflow. The relative humidity and temperature were maintained constant at the levels of RH = 20% and T = 30 °C, and their small variations had no significant impact on the diameters of nanofibers. To form precursor fibers, a 10%wt PAN (Zoltek Co. Nyergesujfalu, Hungary) solution in N,N-dimethylformamide (DMF, from Avantor Performance Materials Poland) was prepared. The process of conversion of PAN nanofibers to carbon nanofibers consisted of two stages, i.e., stabilization and carbonization. The stabilization process was carried out in two stages: first by heating the nanofibers at a rate of 3°C/min to 250 °C and keeping them at this temperature for 30 min, and then by increasing the temperature to 270 °C and holding them for 20 min. The carbonization process was carried out in a tube furnace, both quartz and graphite, in an argon atmosphere (Air Liquid) at several temperatures, i.e., 750 °C, 1000 °C, 1500 °C, 1750 °C and 2000 °C. Heating to the set temperatures was carried out at the rate of 7 °C/min without holding the nanofibers at the final temperature. The samples were named as follows: eCNF750, eCNF1000, eCNF1500, eCNF1750 and eCNF2000. 

After the process of thermal treatment, eCNF was in the form of thin sheets of nonwovens with a thickness of about 20 μm. In order to assess the impact of the thermal treatment process and, thus, the structure of carbon nanofibers on the biological response, it was assumed that the degradation products of this material would have a key impact on the cellular response. For this purpose, most of the research was carried out on carbon nanofibers in a fragmented form, which was intended to simulate the degradation products of this material. In order to obtain the ground form of eCNF, each sample obtained at a different temperature was ground in an agate mortar, changing the material into a powdered form. 

**Figure 12:**
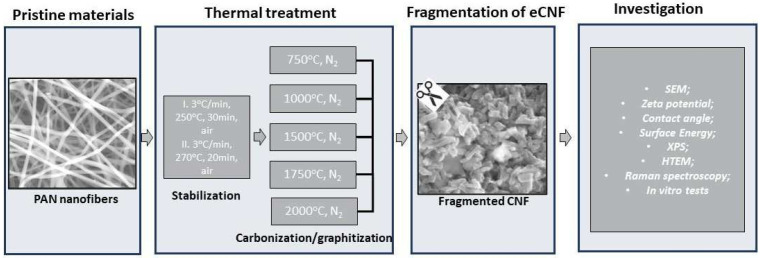
Schematic diagram of the individual stages of preparation and investigation of carbon nanofibers (CNF).

All samples were investigated using a number of methods, namely scanning electron microscopy (SEM) for morphology and microstructure investigation, Raman spectroscopy and high-resolution transmission electron microscopy (HRTEM) for structural investigation, and goniometry for analysis of surface wettability and surface energy. The presence of chemical groups on eCNFs was investigated using X-ray photoelectron spectroscopy (XPS). Chondrocytes (CHON-001 cell line) were contacted with eCNFs at 37 °C for 1 h and 24 h; next, the cells were washed in PBS and analyzed by the comet assay procedure and fluorescence microscopy. The individual stages of sample preparation and testing are presented in the diagram (Figure 12).

### 3.1. Physicochemical Characterization of CNFs

The morphology and microstructure of the CNFs thermally treated at different temperatures in ground form was investigated using SEM (NovaNanoSEM 200, FEI). The SEM images were also used to evaluate the length and diameter of the CNFs after the grinding process using ImageJ software. In total, 100 nanofiber length measurements and 100 diameter measurements were made for each sample. The results were analyzed using statistical software (StatSoft Inc., Tulsa, OK, USA). The Mann–Whitney U test for multiple comparisons was used to evaluate statistical significance at *p* ≤ 0.05. 

The X-Ray photoelectron spectroscopy (XPS) analysis was performed in a PHI VersaProbe II Scanning XPS system equipped with Al Kα X-ray radiation source (1486.6 eV). The measurements were carried out under pressure < 5 × 10^−9^ mbar, with a take-off angle of 45°, and energy passes of 117.5 and 46.95 eV for the survey and core-level spectra, respectively. During the measurements the excessive charge was compensated using Ar+ ions an low energetic electrons. The energy position was calibrated using C1s adventitious carbon peak as the reference (Eb = 285.0 eV). The deconvolution of the peaks and subtraction of background (the Shirley method [71]) were carried out using the PHI MultiPak software. 

Raman spectroscopy measurements were performed using a WITec Alpha 300 M + apparatus with a 600 g/mm mesh, a 488 nm laser and a 50× lens. A total of 10 accumulations with 20 s integration times were recorded for each measurement. Fityk 0.8.0 software was used for the spectra analysis. Spectral deconvolution was performed using the Voigt function [72]. It allowed us to distinguish characteristic bands corresponding to vibrations of carbon structures in samples. The I_D_/I_G_ ratio was determined from the total intensities of the D and G bands as a coefficient describing the degree of crystallinity of the carbons. Additionally, the size of the La crystallite was also determined using the Cançado equation [73]:

La = (2.4 · 10^−10^) · λ^4^ · (I_D_/I_G_)^−1^ (1)

where La is a crystallite size, λ_l_ is the wavelength of the excitation source, and I_D_ and I_G_ are integral intensities of the Raman bands D and G.

A high-resolution transmission electron microscopy (HRTEM) analysis was performed with a Titan Themis 200 kV x-FEG Cs corrected transmission electron microscope. The samples for analysis were deposited from a colloidal suspension onto a carbon coated copper grid. 

The zeta potential (ζ) and the size distribution of the short CNFs were analyzed in buffered saline solution (PBS) using a combination of electrophoresis and the LDV technique (Laser Doppler Velocimetry, Malvern Zetasizer Nano ZS), and with the laser with a wavelength of λ = 520 nm. The range in which the size of fragmented CNFs was measured was from 5 nm to 10 µm. The aim of these studies was to determine how the temperature of thermal treatment affects the size of the degradation products of CNFs as well as their stability in a PBS solution.

The contact angle of the obtained CNF mats was measured by the sessile drop method using a DSA 25E analysis system and KRÜSS ADVANCE 1.6.1.0 software (KRÜSS, Hamburg, Germany). The contact angle was calculated by averaging the results of 20 measurements. The total surface energy (γ) of all types of CNF was calculated using the Owens–Wendt method [74]. In this method, two components of the surface energy are determined, i.e., the polar component (γp) and the dispersion component (γd), using two liquids (water and diiodomethane) with known values of the components γd and γp for this purpose. 

### 3.2. Biological Investigation of CNF on Cell Response 

For estimation of the genotoxicity of the studied eCNFs obtained under various temperature treatment conditions, the chondrocyte cell line CHON-001 (ATCC CRL-2846) was used. The cells were cultured in an MEM medium supplemented with 20% FBS, 1% penicillin-streptomycin, 2 mM L-glutamine and 5% CO_2_ at a temperature of 37 °C. The cells were seeded on six-well plates. The starting quantities of CNF suspensions in PBS were 0.5 mg/mL. For the purpose of homogenization the CNFs in PBS suspensions were mixed for 2 min using an ultrasonic probe (PALMER INSTRUMENTS, Model: CP 130 PB). Two concentrations of the eCNF suspension, 21 µg/mL and 42 µg/mL, were added to a wells containing cells in 0.5 mL of culture medium. Then, the chondrocytes were treated with all types of CNFs at 37 °C for 1 h or for 24 h. After treatment, the cells were washed in PBS, and analyzed by an alkaline version of the comet assay procedure presented in the source publication [75] as well as described in one of our previous publications [57].

Chondrocytes were lysed for 1 h (1% Triton X-100, pH > 13). Next, the alkaline electrophoresis (30 min, 6 °C, 30 V and 300 mA) was performed. The cellular DNA was stained with ethidium bromide (17 mg/mL). The epifluorescence microscope (Olympus BX-50, Tokyo, Japan) with the Komet 3.0 software (Kinetic Imaging Company, Liverpool, UK) was used to DNA damage visualization and analysis. A quantitative estimation of the DNA damage was performed using the T-DNA parameter (DNA percentage in the comet tail). Additionally, the dead cells were manually counted in all samples. The level of DNA damage was assessed by measuring 200 cells.

In order to assess the cytotoxicity of eCNFs, the method of simultaneous staining of living and dead cells with two fluorescent dyes, i.e., calcein AM (C1359, Sigma-Aldrich, Hamburg, Germany) and propidium iodide (P8464, Sigma-Aldrich, Germany) was used. Calcein AM crosses the cell membranes of living cells and stains them green, while propidium iodide stains cells with a damaged cytoplasmic membrane red. In total, 20,000 CHON-001 cells in chondrocyte growth medium CGMTM (Lonza, catalog number: CC-3216) were seeded into each culture well. Cells were grown on eCNF after fragmentation for 72 h and stained with the fluorescent dyes mentioned above. The assessment of changes in cell morphology in contact with eCNFs was performed on the basis of fluorescence microscopy images (Zeiss Axiovert 40, Carl Zeiss, Oberkochen, Germany).

## 4. Conclusions

In this study, comprehensive studies of the microstructure, surface chemistry and degree of crystallinity of fragmented electrospun carbon nanofibers obtained under various conditions of thermal treatment in the temperature range of 750–2000 °C were carried out. The aim of these physicochemical and structural studies was to evaluate the influence of these materials on the biological response in contact with the chondrocyte cell line (CHON-001 cell line), in particular, to assess the cytotoxicity and genotoxicity of these materials using the comet test. The results of the Raman spectroscopy and HRTEM studies showed changes in the crystallinity and degree of ordering of carbon nanofibers with an increase in the temperature of the thermal treatment. In contrast, the increase in the carbonization temperature leads to the reduction in functional groups related to both oxygen and nitrogen, as evidenced by the results of XPS tests. The nanofibers obtained at above 1000 °C, e.g., eCNF1500, eCNF1750 and eCNF2000, showed the complete removal of the nitrogen-related groups and a significant reduction in the oxygen-related functional groups. The increase in the degree of crystallinity of the samples affects the size of the degradation products, which decreases with increasing temperatures. In turn, the removal of chemical groups associated primarily with oxygen is reflected in the decrease in wettability of these materials. The results of genotoxicity tests performed with the use of the comet test showed significant differences in the biological response, which were strongly dependent on the degree of thermal treatment of the tested nanofibers. As shown by the results of the comet test, the genotoxic effect is demonstrated by nanofibers that were subjected to thermal treatment at temperatures of 750 and 1000 °C. The summary of the results of structural and microstructural studies as well as the analysis of the chemistry of these materials allowed us to answer the question of which of the material factors is responsible for the genotoxic effect. The results of our research have shown that the increase in the degree of crystallinity, related directly to the increase in the I_D_/I_G_ parameter, does not have a negative impact on the biological response, similarly to the degradation products of these materials, which are characterized by much smaller dimensions than the nanofiber degradation products obtained at low temperatures, i.e., eCNF750 and eCNF1000. Other parameter that may affect the cellular response is the wettability of the surface, which in the case of the tested samples decreases with increasing temperature of the thermal treatment of nanofibers. Generally, cells prefer hydrophilic surfaces to a greater degree than hydrophobic ones; therefore, this parameter indicates that cells should prefer eCNF750 and eCNF1000 nanofiber surfaces, which as it turns out, from the point of view of cell viability, is not very important, while the genotoxicity for these materials is higher. Considering all factors, the most likely inducer of a genotoxic response is the presence of residual nitrile groups in the eCNF750 and eCNF1000 samples causing chromosomal aberrations and the exchange of sister chromatids.

## Figures and Tables

**Table 1:** Elemental composition from XPS analysis of CNFs after various thermal treatments.

Materials	Atomic Concentration (at.%)		
	**C**	**O**	**N**
eCNF750	85.4	3.2	11.5
eCNF1000	91.6	5.4	3.0
eCNF1500	98.7	1.3	-
eCNF1750	99.4	0.7	-
eCNF2000	99.6	0.4	-

**Table 2:** Zeta potential results of the eCNF samples in a PBS solution at pH = 7.2.

Sample	eCNF750	eCNF1000	eCNF1500	eCNF1750	eCNF2000
Zeta potential ζ (mV)	−17.0	−20.5	−22.5	−21.9	−19.4

## Data Availability

The data presented in this study are available from the corresponding author upon request. At the time that the project was carried out, there was no obligation to make the data publicly available.

## Supplementary Materials

The following supporting information can be downloaded at: https://www.mdpi.com/article/10.3390/ijms23116278/s1.
