# Supplementary material for: Influence of Heat Treatment of Electrospun Carbon Nanofibers on Biological Response
Source: Int J Mol Sci. 2022 Jun 3;23(11):6278. doi: 10.3390/ijms23116278 (PMC9181356; doi:10.3390/ijms23116278)
Supplement: Supplementary file 1 [file ijms-23-06278-s001.zip › ijms-1758464-supplementary.pdf]

Supplementary material

Influence of heat treatment of electrospun carbon nanofibers on biological response

Jaroslaw Markowski[1], Marcel Zambrzycki[2], Wojciech Smolka[1], Agnieszka Panek[3], Maciej Gubernat[2], Pawel Czaja[4], Mateusz Marzec[5], Aneta Fraczek-Szczypta[2*]

[1]Laryngology Department, School of Medicine in Katowice, Medical University of Silesia in Katowice, Poniatowskiego 15, 40-055 Katowice Poland; wojciech.smolka@op.pl (W.S.); jmarkowski@sum.edu.pl (J.M.)

[2]Faculty of Materials Science and Ceramics, AGH University of Science and Technology, Mickiewicza 30 Av., 30-059 Krakow, Poland; zambrzycki@agh.edu.pl (M.Z.); Maciej.Gubernat@agh.edu.pl (M.G.); afraczek@agh.edu.pl (A.F.S.)

[3]Institute of Nuclear Physics, Polish Academy of Sciences, Radzikowskiego 152 St., 31-342 Krakow, Poland; agnieszka.panek@ifj.edu.pl (A.P.)

[4]Institute of Metallurgy and Materials Science, Polish Academy of Science Reymonta 25 St., 30-059 Krakow, Poland; czaja.p@imim.pl (P.C.)

[5]Academic Centre for Materials and Nanotechnology, AGH University of Science and Technology, Mickiewicza 30 Av., 30-059 Krakow, Poland; marzecm@agh.edu.pl (M.M.)

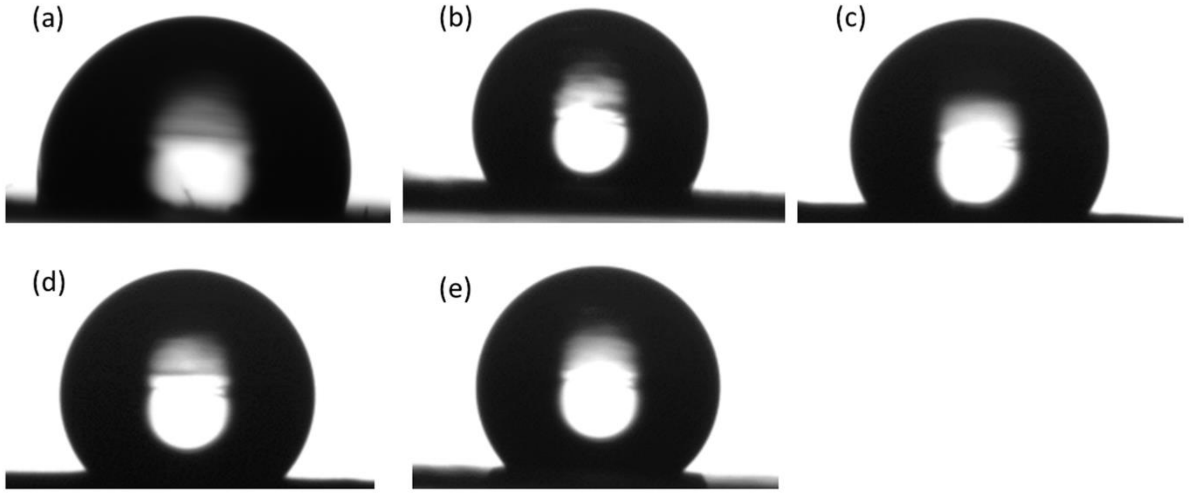

Figure S1. Imagines of contact angle in contact with (a) eCNF750, (b) eCNF1000, (c) eCNF1500, (e) eCNF1750, (e) eCNF2000
